# Developing and implementing national health identifiers in resource limited countries: why, what, who, when and how?

**DOI:** 10.1080/16549716.2018.1440782

**Published:** 2018-03-05

**Authors:** Eduard J. Beck, J. Mark Shields, Gaurang Tanna, Gerrit Henning, Ian de Vega, Gail Andrews, Philippe Boucher, Lionel Benting, Jesus Maria Garcia-Calleja, John Cutler, Whitney Ewing, Boonchai Kijsanayotin, Tapiwanashe Kujinga, Mary Mahy, Keletso Makofane, Kim Marsh, Chujit Nacheeva, Noma Rangana, Mary Felissa Reyes Vega, Keith Sabin, Olga Varetska, Steven Macharia Wanyee, Stephen Watiti, Brian Williams, Jinkou Zhao, Cesar Nunez, Peter Ghys, Daniel Low-Beer

**Affiliations:** a LAC RST, UNAIDS, Georgetown, Guyana; b Health Informatics Consultant, Verona, WI, USA; c National Department of Health, Pretoria, South Africa; d Health System Technologies (Pty) Ltd, Cape Town, South Africa; e Department of Health, Western Cape Government, Cape Town, South Africa; f Global Health Observatory, WHO, Geneva, Switzerland; g Department of the Premier, Western Cape Government, Cape Town, South Africa; h HIV Department, WHO, Geneva, Switzerland; i University of North Carolina at Chapel Hill, Chapel Hill, USA; j Health System Research Institute, Bangkok, Thailand; k Pan-African Treatment Access Movement, Harare, Zimbabwe; l SIM Department, UNAIDS, Geneva, Switzerland; m Anova Health Institute, Johannesburg, South Africa; n Ministry of Public Health, Bangkok, Thailand; o Treatment Action Campaign, Johannesburg, South Africa; p Ministerio de Salud, Lima, Peru; q International HIV/AIDS Alliance, Kiev, Ukraine; r IntelliSOFT Consulting Limited, Nairobi, Kenya; s Community Health Alliance, Kampala, Uganda; t Stellenbosch University, Stellenbosch, South Africa; u Global Fund, Geneva, Switzerland; v LAC RST, UNAIDS, Panama City, Panama

**Keywords:** National health identifiers, resource limited countries, health-information systems, confidentiality security personal health information, person-centred care

## Abstract

Many resource-limited countries are scaling up health services and health-information systems (HISs). The HIV Cascade framework aims to link treatment services and programs to improve outcomes and impact. It has been adapted to HIV prevention services, other infectious and non-communicable diseases, and programs for specific populations. Where successful, it links the use of health services by individuals across different disease categories, time and space. This allows for the development of longitudinal health records for individuals and de-identified individual level information is used to monitor and evaluate the use, cost, outcome and impact of health services. Contemporary digital technology enables countries to develop and implement integrated HIS to support person centred services, a major aim of the *Sustainable Development Goals*. The key to link the diverse sources of information together is a national health identifier (NHID). In a country with robust civil protections, this should be given at birth, be unique to the individual, linked to vital registration services and recorded every time that an individual uses health services anywhere in the country: it is more than just a number as it is part of a wider system. Many countries would benefit from practical guidance on developing and implementing NHIDs. Organizations such as ASTM and ISO, describe the technical requirements for the NHID system, but few countries have received little practical guidance. A WHO/UNAIDS stake-holders workshop was held in Geneva, Switzerland in July 2016, to provide a ‘road map’ for countries and included policy-makers, information and healthcare professionals, and members of civil society. As part of any NHID system, countries need to strengthen and secure the protection of personal health information. While often the technology is available, the solution is not just technical. It requires political will and collaboration among all stakeholders to be successful.

## Background

**Figure 1.**
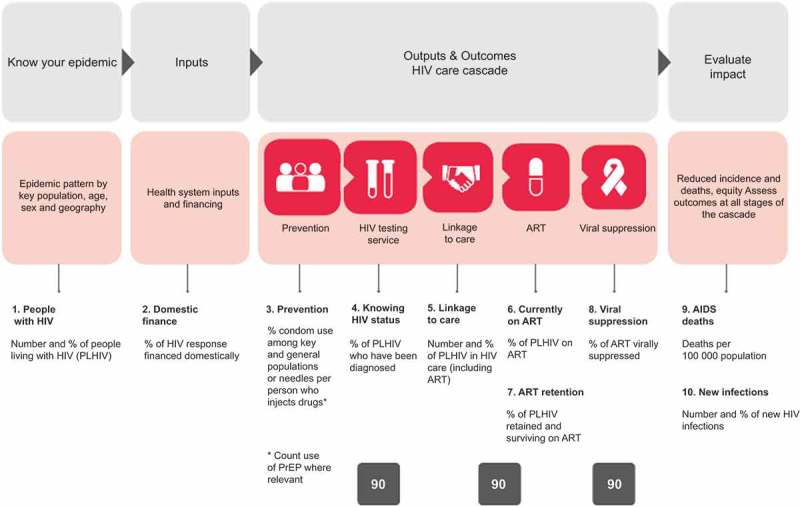
Ten WHO indicators assessing the ’90–90-90’ program showing linkages with other sources of data including case based surveillance and patient monitoring.

The ‘HIV Health Sector Cascade’ framework was recently introduced to monitor and evaluate a country’s HIV Response (Figure 1). This framework was first developed at CDC [1] and has since been developed in many countries, and forms the basis of the UNAIDS ‘90–90–90’ targets [2]. Similar frameworks are now being applied to HIV prevention [3], to specific sub-populations enrolled in particular programs [4], and to other communicable [5] and non-communicable diseases (NCDs) [6].

The framework tracks the use of different services by individuals and populations across disease categories, time and place, and aims to link information from a range of different sources, including vital statistics, person-centred patient monitoring and case surveillance (Figure 1) [7,8]. As individuals are tracked across different services, their information can be linked to develop longitudinal medical records. De-identified individual level information collected can also provide policy-makers, clinicians, PLHIV and other stakeholders with information to monitor and evaluate health services at facility, sub-national and national levels [9].

The implementation of anti-retroviral (ART) programs has increased the number of PLHIV in many resource limited countries, as their life-expectancy on ART approximates that of people not living with HIV, provided life-time ART is started with a CD4 count greater than 500 cells/mm3 [10]. The World Health Organization (WHO) now recommends that starting ART at time of diagnosis [11] and that will result in increased number of PLHIV aged 50 years or older [12]. The most common co-morbidities of older PLHIV are non-HIV cancers, cardiovascular disease and other NCDs, and these are also their commonest cause of death [13]. PLHIV will therefore increasingly need to use non-HIV services, especially in countries with successful HIV responses. To monitor and evaluate the HIV-response in these countries, the use, cost, outcome and impact of NCDs’ services used by PLHIV will also need to be tracked, especially as integrated HIV and non-HIV services are likely to be more cost-effective [14]. Such integration can contribute to the development of services for other chronic conditions in resource limited countries, especially as NCDs are an important cause of morbidity and mortality in their own right [15].

The use of health services within a country should be tracked across facilities and the personal health information linked over time as part of providing universal health coverage [16]. Countries increasingly recognize this need; while some still focus only on developing their HIV Cascade, other resource limited countries now recognize the need to develop and implement their HIS across all healthcare sectors. While Zambia has developed an extensive HIV specific cascade [17], AMPATH in western Kenya, which started as an HIV system, has now been expanded into a network of facilities that provides health services for a range of other diseases including HIV [18]. Some countries are even more ambitious and want to link healthcare information with information obtained from insurance systems, social services or other societal sectors, as has been developed in Denmark [19].

To ensure optimal service delivery across such a variety of sites, specific identifiers need to exist which link all information to that individual [20]. In health facilities, *facility-identifiers* are alpha-numeric codes used to identify an individual within that particular facility. Within facilities, different clinics may have their own identifier, *clinic-identifiers*. Several facilities can be linked with each other to provide services through a particular program, for instance a tuberculosis (TB)-program. Individuals who are part of that program can have specific *program identifiers* that track the use of services by that individual within the program. However, neither *facility*- nor *program-identifiers* can be used outside the specific facility or program, and the use of services used outside the facility or program cannot be tracked and data not linked. To link these data an identifier needs to capture all healthcare services; a *national health identifier* (NHID). NHID should be universally unique within the country’s health system, is assigned to an individual for life, is never used for another individual, and can be linked to track an individual through their health journey as they use services at different facilities or within different programs [20]. The five basic functions that a NHID should support and its seven core elements are described in Box 1.

**Box 1.** The five basic functions and seven core elements of a NHID [20].

The five basic functions that a NHID should support are: ability to unambiguously identify individual patients at all care settings, from a clinical and administrative perspective; ability to link a variety and continuously evolving set of data elements across institutions, service providers and time, to constitute a lifelong view of the patient’s medical history, needed to deliver healthcare; ability to aggregate information across institutional boundaries for specific services and health outcomes, to track health program implementation and strengthening surveillance; protect the privacy, confidentiality and security of personal health information at the site where the information is held and by de-identification of records before they leave the primary site; reduce operational costs by supporting automated record management and information sharing.
The seven core elements of a NHID, required for the system to perform its functions consist of: an identifier system, consisting of alphanumeric characters with check-digit functionality, ensuring that numbers or characters are not transposed when typed, while the identifier should exclude characters that represent any aspect of the individual’s identity, including date of birth, gender or other personal identifiers identification information, including demographic or biometric data cross-references to local, site- or program-specific health identifiers – like facility medical record numbers or TB program identifiers – and other identifiers – including, civil identifiers, national identity documents, passports, drivers’ licenses, or others mechanisms to hide or encrypt identifiers so they don’t disclose any personal information software to register patients software to search, match, merge, encrypt or otherwise manipulate underlying information an administrative infrastructure, including human and financial resources that comprises the governance centre for managing and controlling the how NHID’s are allocated and used.

## Paper, power and network: three tier developmental infrastructure

While the development and implementation of NHID are facilitated by the existence of reliable electricity supplies in health facilities, in low- and middle-income countries many facilities may have no or limited access to electricity, computers or the internet; the presence or absence of electricity is a major defining factor. As infrastructure develops, health centres undergo technology transitions and three tiers of permanent facilities are recognized, while transient health posts and community-based care comprise Tier 0 facilities:

*Tier 1 – Paper*: facilities with no reliable power or telecom; cold chain may be burner powered.

*Tier 2 – Power*: facilities with a minimum of reliable daily power – solar, generator, power lines and uninterrupted power supply (UPS) – sufficient to charge/operate an efficient computer.

*Tier 3 – Network*: facilities with reliable daily telecommunications and power, at least sufficient to have certain deferrable clinic operations normally depended on internet-based applications.

*Tier 1 health facility*: operations provide continuity of care for patients across the system platform but for specific clinical programs, using semi-durable and portable paper documents. Familiar examples are ante-natal clinic (ANC) cards, delivery records, under-five cards for tracking growth and immunizations in children, and TB cards. South Africa has a *‘Road to health chart’* [21] that is used to track child care and is a requirement for a child to be enrolled in primary school. Most clients who need them carry them for the duration of using the service. Each of these tools provides a degree of continuity within the scope of a single protracted service. Each program may issue its own identifier for the duration of the episode of care and life of the paper documents: primary care records are usually collected in free form text.

Some countries have developed documents or ‘health passports’ which integrate some program specific information with general primary care records [22] but they have a finite capacity before needing to be restarted with a blank document. These are used across health programs and kept by patients. In other situations, clients may be expected to provide their own paper document, perhaps a student exercise book, for recording the clinical visit, and there is variability whether the facility or client retains the document.

Finding older paper documents may be difficult to do with consistency; after a certain number of years, old documents are archived or discarded to make room for new. Filing and retrieval may be improved by using a single standardized identifier; however, sharing a single physical copy record can introduce contention between programs due to their specific needs. Whether integration of a single physical record per facility might reduce the small degree of within-program discontinuity that years of paper operational experience produced, would need to be assessed and might depend on the filing logistics and size of the facility. The consensus is, however, that the use of a unified, cross-programmatic identifier is a pre-requisite to improve continuity of care across health services within as well as between health facilities.

Providing good long-term or complex care with paper records, without fax, copier or good paper supply, is quite hard but an important first step for linking records. If a client is mobile, continuity of care generally fails due to paper record sharing difficulty, not for lack of an NHID. Fixing these issues with Tier 1 resources is sometimes more difficult and less fruitful than efforts to get electricity. Sites need power to most effectively and efficiently implement the continuity of care purpose of a NHID.

*Tier 2/3 – Electric facilities*: due to the critical mass of a changing national economy, growing population or political developments, a more reliable electrical infrastructure with some excess capacity may be achieved, and the paper facility transitions to a Tier 2 – electric facility. This is the *biggest* and most enabling infrastructure transition.

**Figure 2.**
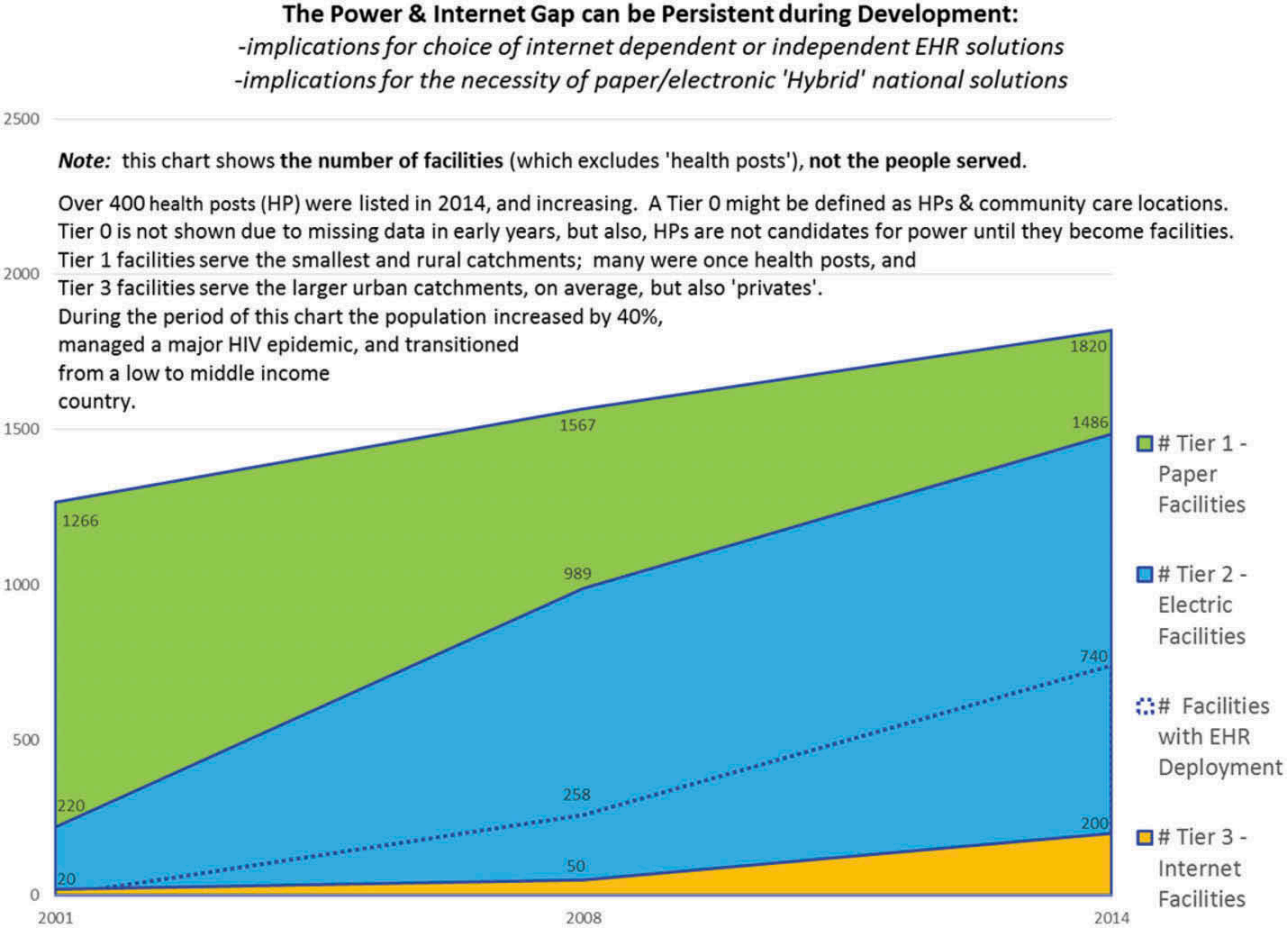
The transition from Tier 1 to Tier 3 facilities, sub-Saharan country 2000–2014.

In Zambia, 220 out of 1138 (19%) Tier 1–3 health facilities in 2000 had sufficient power for a simple decentralized electronic health record (EHR) with one computer, printer, universal serial bus (USB) smart card reader, and UPS. This number increased to 989 out of 1781 (56%) in 2008, and to an estimated 1200 out of 2405 (50%) facilities with basic electricity by early 2014. During this same period the population grew from 10 to over 14 million people (Figure 2).

While a Tier 1 ‘paper’ facility might hope to have developed to Tier 3 in 5–10 years, this does not mean that in just 5 years there will be no need for a hybrid system, or that the country should plan to upgrade immediately to an internet dependent EHR solution. If a country has a large number of Tier 1 and 2 facilities, an internet dependent design – Tier 3 – may not be optimal. Deployment of internet dependent solutions might block a large percentage of the population from receiving good continuity of care that can be delivered without the internet, for instance via a smart card held by the client. The latter can be used to store essential elements of any health records, along with identifier and quasi-identifier authentication elements.

Although an internet-based solution may provide added value in some clinics as the infrastructure develops, additional and more remote clinics may be built as a country’s population grows, that will often start as a Tier 1 paper facility before developing over time Tier 2 electrical capacities. However, even in fully electronic environments, paper ‘back-ups’ may be required. When there is a down-turn in the economy, infrastructure development may stall or even regress, and systems need to continue to function during hard times. In addition, systems should be designed that continue to function during natural or man-made disasters.

## Developing and implementing a NHID and NHID-system

**Figure 3.**
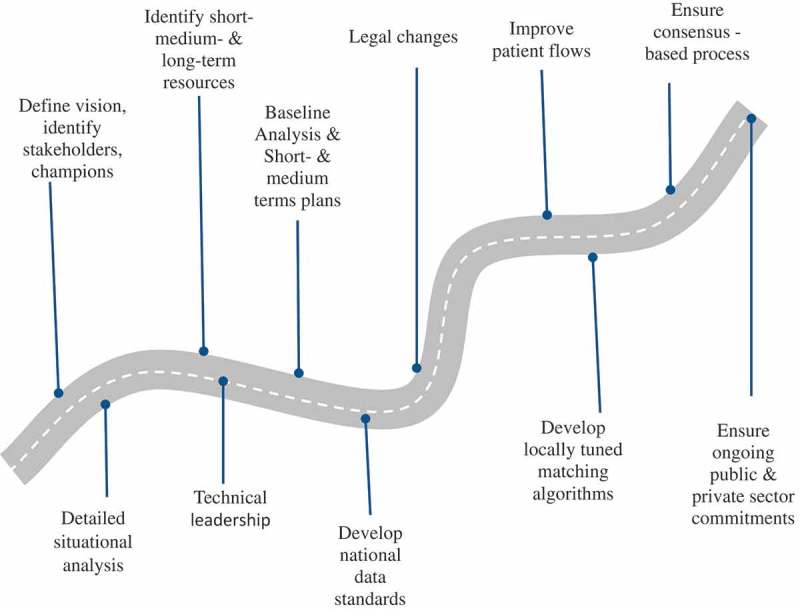
Initial steps to develop a NHID system.

The development and implementation of a NHID is a system-wide process and to be successful the first step is *carry out a comprehensive assessment* (Figure 3). Early in this process a group of dedicated stakeholders should be brought together, who will start to define the vision for the country. All stakeholders that need to be involved in the process and are willing to act as medium- to long-term champions, need to be identified and engaged. This process also needs to provide a technical review of the health-information system operative in the country, drawing on all available information including secondary sources.

The forms currently in use by the Ministry of Health and the data currently routinely collected by them need to be reviewed and assessed. Similarly, the key health service issues need to be identified and the extent that better information can address them, collected through a better information infrastructure or updated laws. The uses of a NHID need to be circumscribed as how they contribute to improvement of services and a decision made whether to focus on the public sector or also include the private health sector. An inventory of existing identifiers used in the country by major systems needs to be drawn up, their use and structure described as well as that of quasi-unique identifiers used in these same systems. Existing record linkage and de-duplication efforts also need to be identified. For example, one approach could be to consolidate the various systems, making use of matching algorithms to allocate NHIDs to existing information, and then use the new NHID to go forwards. It is important to consider legislation that governs existing identifiers and appropriate control of access to information. This will provide useful input into understanding governance arrangements between the authorities, using existing identifiers to link, access and use the information.

A baseline appraisal of the existing national health informatics infrastructure needs to be made, including the review of past assessments and secondary data (Box 2). The existing separate health-information systems in the various facilities need to be listed, the modules they support and users’ experiences with them. The strength of the development teams of any of the commercial systems needs to be assessed and the costs of integration, data conversion and scaling up to country level need to be explored. The top 5–10 systems in chosen sectors need to be explored in depth, to be able to identify which will become the inventory of health information standards and systems used in the country. The ownership of the information, which is created by linking data from the health sectors, needs to be agreed on.

**Box 2.** Baseline assessment of existing national health information infrastructure.

Such a survey needs to include the following: a review of previously performed assessments such as Health Metric Network [23,24] or other surveys and secondary sources the number of facilities with and without with electrical power, with internet and percentage of ‘down time’, and the number of anticipated future new facilities an assessment of what power and telecom networks exist, what is being planned and time frames, checking estimates with internet service providers and national electrical and telecommunications provision identification of those parts of the country that are inaccessible during the year and how this affects power or telecommunications. an assessment of the number of facilities with existing computers, computer users and their skill levels, the presence of air conditioning and secure room for a server and identify the number of facilities with electronic medical or health records list and characterize each different EHR initiative at the level of data structures used and validations applied to the collected data.

Information technology professionals need to be identified for software development, database administration and networking, including health informatics and system analysts. In addition, persons who have experience in record linkage for multimillion record files also need to be identified. Ensure that software from vendors are used that can generate NHIDs based on local requirements and any vendor solutions should be owned by the country to avoid being locked in to any one vendor. Alternatively, ensure links to NHIDs generated by third-party systems can adapt to changing requirements. Identification of the right software systems, vendors and support infrastructure are key considerations, as these are part of an ever-evolving environment.

Identify or develop staff who can supervise the use and quality of data being collected, and analyse the data to provide feedback on the evolving structure of service provision. Find the best academic programs and talk to relevant academics while keeping abreast of new developments. Assess existing laws, regulation and policies, including human rights protection, information ownership and stewardship, information standards, accountability and governing authorities. Assess public perception, trust and buy-in for the vision of a NHID, popular conceptions or misconceptions. Assess existing privacy, confidentiality and security guidelines, and their implementation in the health sector in terms of data collection, access, storage, transfer, use, disposal and data stewardship. Based on this assessment, the process of changing any necessary legal and statutory requirements to deposit personal health information in data warehouses or repositories can commence. In parallel, one needs to make an assessment of operative administrative business processes and improve these where required and if possible.

### Define the vision and the resources required

Focus on the benefits to the whole system that the NHID enables in terms of healthcare in a country and not on the actual NHID implementation. Reflect the sense of the needs and ambitions in the country that were uncovered during the assessment. It should be produced and owned through a group effort, including some key people who are committed to the vision they formulate. Find champions for the vision who are socially accepted, persistent and committed. These should be sought among clinicians, other healthcare professionals, academics, civil servants, politicians and community leaders. Share and sell the vision and try to link such a long-term project to multi-party support and not one political platform, while recognizing and highlighting the benefits obtained from each progressive step towards achieving the vision.

Identification and assessment of resources is a continuous process of developing and implementing large, multi-year, national systems efforts. Evaluate available resources in terms of funding, technical infrastructure, information, communication and technology, human resources, including technical, legal, political and civil society leaders. Different resources will come to bear for different areas and stages of the implementation. Similarly, different benefits will come to bear for different stakeholders at the various milestones during the implementation of the system. It is important to identify those and work according to strict timelines to satisfy expectations. Submit relevant *grant applications*, get *public and private sector commitments*, including industry, whether for technical assistance, logistics, data for testing or infrastructure development commitments. Developing working relationships with relevant academic colleagues and institutions is very important in this context. The most critical resource is enduring political will and strengthening of the information communication technology (ICT) infrastructure needs to be prioritized as part of the national HIS strategy.

### Develop a short-term and medium-term plan

Various parallel processes will need to be planned, integrated and implemented during the first few years. *Development of national data standards* needs to be started very early, based on international standards and involving the colleagues and champions identified so far. Multiple information systems need to interchange data with each other and systems must send and receive data conforming to standard definitions, standard codes and standard field lengths. These are of fundamental importance to data quality and integrity, and should be used by staff of all disciplines, particularly those involved in the collection, processing and analysis of information. This involves the development of a single definitive national inventory of information on health and possibly social care datasets, and incorporates a national data dictionary, information on the existing national datasets and new developments. Such single inventory – called the *National Data Catalogue* in Scotland [25] – would:

1. Improve provision of comprehensive metadata on national data through a single point of access.
2. Eliminate duplication of information.
3. Enhance support to research on health and social care.
4. Encourage greater use and awareness of national data standards and definitions to improve the quality of information available to support health and social care services.

The data dictionary is a one-stop shop for health- and social-care data definitions and standards, and should be maintained by a single agency. As new definitions and standards are agreed they will be published on the data dictionary site. The national dataset section contains the full list of datasets held nationally, along with information about the datasets, such as size of dataset, population coverage, overall data quality, as well as publications that use these datasets and other general useful information [25].

These processes need to be driven by established governance structures that are supported by the political leadership to complete them, since it will involve making many changes, including to existing systems. A strategy needs to be developed in terms of the use of personal health information and, as the quality and quantity of this information increases, such information is increasingly used to monitor, evaluate and improve services.

All required *changes in law* need to be detailed such that personal health information will be safe for all in the future. The technicians need to find test data for *developing locally tuned matching algorithms* and learning what quasi-identifiers are needed in the country, what the other existing information systems do and whether they work well. Work with healthcare professionals to *draw up patient flow charts* and where possible improve work flows. Map out how data are captured and used throughout the information system. This information can be used to model how the information technology can facilitate this process and will also show how data should be exchanged between various vertical systems as data objects. Software such as *Business Process Model and Notation* 2.0 may provide useful tools for this purpose [26]. If matching algorithms are to be used that worked well for smaller number of individual files, they may not necessarily be adequate if they are used for a larger number of files.

A need exists to reduce the number of clinical and patient based administrative systems information systems to a few systems, either by executive order or through some competitive process that objectively assesses existing systems according to agreed functionality and compliance with standards that are able to converge into a standard in a reasonable time. For instance, unique identifiers are being produced and used for programs for key populations in various countries [27]. The greater the number of existing nonstandard systems, the slower the plan should be developed and implemented, and the greater the importance of building *consensus*. The vision needs to be discussed publicly, including the benefits and risks. Developing consensus is an iterative process. Different applications can theoretically work the same electronic health record data structures, and countries may in some situation maintain the services of the vendors of these different applications. However, this is not possible if multiple incompatible data structures exist in the country.

As the correct engineering and design start to evolve, they will need to be discussed regularly with technical experts, champions, lead users, political leadership and civil society. Developing and implementing the relevant legal framework is likely also to be a multi-year process. An incremental process provides time to pass laws, allows confidentiality and security issues to be considered carefully, and ideas can be tested at small scale. Civil society needs to know that the risks are not zero and understand their parts in keeping their information safe. Many interests will arise from the project and one needs to plan how to build the largest and most durable consensus: who gets the initial contracts, the contingencies for retaining the contracts, who owns the source code or to what extent is it ‘open source’, where pilots are to be performed, which organizations will be involved in the training, and who will be data stewards, if not already defined.

Usually more than one way to solve a problem exists, whether to build software or infrastructure. Learn about different opinions, understand them and persuade key individuals until congruence begins to emerge. Persistence and good will may bring design and engineering consensus on the same or faster time frame as establishing the national information standards, the necessary legal infrastructures and securing resources.

## Planning what to do

The design of the NHID and system will need to address the issues raised by the assessment.

**Figure 4.**
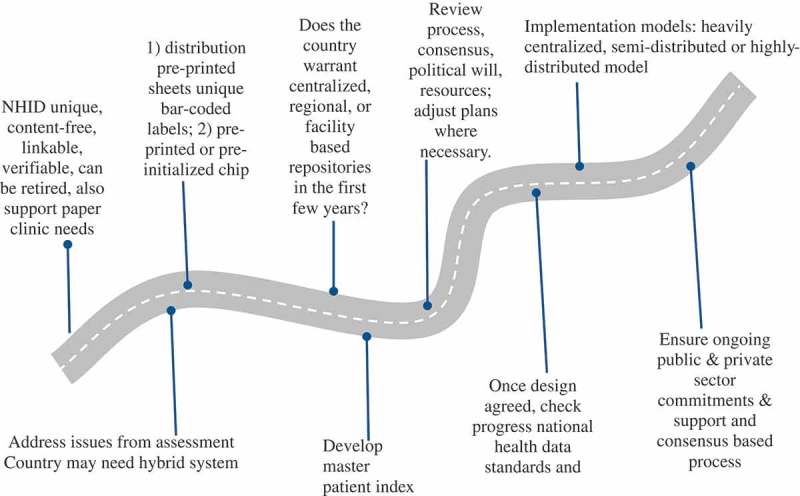
Developing and implementing NHIDs and sections of the NHID system.

Significant design choices will still need to be made. The NHID itself will need to be unique, focused, assignable, accessible, atomic, content-free, usable, concise, linkable, unambiguous, can be verified and validated, retired, retroactive, and public in order to also support paper clinic needs (Figure 4). Use information from the algorithmic testing and the assessments of the enrolment processes of the various extant clinical information systems, to help solidify the minimum quasi-identifiers and biometric selection consensus. Given the existence of Tier 1 clinics, it is likely that in many countries a hybrid system will be needed.

Considering the infrastructure in the country, will it be possible to do central assignment or does one need to rely on distributed assignment? Aside from ‘blocking’, two reasonably practical types of central assignment can work without full network coverage. First, the distribution of pre-printed sheets of unique bar-coded labels to paper clinics, for subsequent pasting on the clinic documents, laboratory orders and prescriptions of a patient in paper contexts. The bar-coded identifier can also be used in automated settings, as for instance is done in South Africa and Malawi. This does require keeping supply lines full as the label sheets are used. One needs to pilot it to confirm this is workable with existing logistics. Second, the issuance of pre-printed or pre-initialized chip card identifiers, which can be both used manually in paper clinics and automatic with no manual look-up – in electronic clinics. In either case, the bar-coded identifier will work in both paper and electronic clinics as the public identifier.

In the Western Cape, South Africa, the distribution of pre-printed sheets of unique bar-coded labels to paper clinics works well, but there must be a single source system generating these, the *Master Patient Index* (MPI). Cheaper alternatives to the more expensive chip cards are available and have been found to work well in more stationary populations, including the use of laminated plastic cards with QR codes or bar codes. However, non-chip solutions exclude the possibility of carrying the digital health record data with the card identifier. The chip card provides an inter-facility continuity of care benefit to the client in Tier-2 non-network environments that competes with Tier-3 infra-structure of care. Giving bar codes on a sticker to a patient to bring to the clinic at the next appointment can also work well for identification and linkages at one clinic, but does not consolidate data for providers or enable inter-facility continuity of care. All of these solutions, however, require electricity to be available in the facility.

Use a system that is designed to be a population-oriented EHR that is proven to be interoperable, can handle large volumes, does not slow down the process and is proven to be robust. Consider whether, based on the size of the country, mobility and telecommunications limitations, the extent that country focuses on developing centralized, regional or facility based repositories in the first few years when the repositories are quite limited for producing aggregate indicator data or are full EHR repositories. Any Tier 1 solution will require physical transport of paper-based data from remote places with monthly update cycles, until power is deployed and the new Tier 2 facility has access to electricity. For such Tier 2 or 3 facilities, one model is to keep some or all data from EHRs in facilities or districts in compressed and encrypted format on a chip card, as has been introduced in Zambia [28]. Information will be accessible to clinicians in facilities for patient care but at the district level, confidential health data are used only for aggregate public health reporting and identifiers used only for record linkage and de-duplication. The size of the country and the percentage of Tier 1 facilities affect the difficulty with quasi-unique identifiers-based de-duplication and matching.

Review previous steps in the process, adjust plans where necessary, and confirm one has kept project consensus. Review resources required now that one can project the cost of the design given the type of technical resources needed. Ensure the availability of adequate funding and other resources based on the existing consensus and political will. Once a design has been agreed on, check that progress has been made on national health information standards and legal updates. If not, work on those areas that have weakened. Check with leadership to see if an executive decision may be forthcoming, including on what to do with incompatible systems.

There are a number of different models for implementing NHIDs, which vary in the number of service points, capacity to scale up and provide national services, complexity and costs. Box 3 describes three models as examples of the many variations that should be considered [20].

**Box 3.** Different models for implementing a NHID [20].

***Heavily centralized model***. This model typically leverages existing government services that occur at one central location, such as the capital city. Characteristics of a heavily centralized model include the following: Costs are minimized since the model can use enrolment locations that are already providing registration services and possibly are already issuing permits or licences. Typically, a single or small number of events induces the citizen to apply for inclusion in the identification system. These events might include birth, reaching a certain age, hospitalization, joining the military, or applying for a driving or marriage licence. The model lends itself to centralized administration and issuance of bar-code stickers or cards. Annual costs are typically lower than for other models due to the limited number of people applying on an annual basis, and use of existing infrastructure to receive applications. The costs of processing applications and administrative costs of the national registry are nearly the same per enrolee for all models.
If the country has predominantly more Tier 1 facilities instead of Tier 2 or 3, one has to solve the logistic issues of having a printer in the capital serve the label printing needs of all the rural clinics in the country, something that can be insurmountable. A compromise between highly centralized and highly distributed Tier 2 or 3 facilities is to print bar codes on initialized chip cards centrally, thereby assuring their uniqueness, so that the identifier on the chip equals the printed bar-code. Such cards can be centrally produced but locally assigned to clients.
***Semi-distributed model***. This model is similar to the heavily centralized model, but it leverages existing government services that typically occur at regional or provincial centres. Characteristics of a semi-distributed model include the following: Costs are typically somewhat higher than for a heavily centralized model but lower than for a highly distributed model. Typically, a single or small number of events induces the citizen to apply for inclusion in the identification system. These events are similar to those for a heavily centralized model. Typically, the application points have card printers and can issue cards upon administrative approval.
***Highly distributed model***. In this model, NHID administration occurs at the point of health service delivery throughout the country. Characteristics of a highly distributed model include the following: There is a much more complex rollout schedule, with a number of teams performing site readiness to meet an aggressive schedule, such as 1–2 years. Costs are higher due to the higher number of application and issuance points and the number of teams executing site start-ups. If an aggressive schedule is desired, a higher number of rollout teams will be required, and administrative costs will be higher in processing the increased number of applications. Many events will typically induce the citizen to apply for inclusion in the identification system, including all of the previously mentioned events plus community enrolment efforts and medical events such as hospitalization or clinic visits. A carefully thought-out and available communication system is required, with robust connectivity to at least the district level and probably also the larger site level. Rollout costs will be higher due to a larger rollout team, which includes providing equipment, hiring staff and training on the identification system. Unless there is a uniform national language, translation will be needed at least for the application forms and possibly also for the data-entry screens. This is a more complex NHID registry service model, due to the need to communicate to a wide variety of electronic systems, provide printouts for paper systems, and maintain highly synchronized pools of data to ensure no single point of failure in the national system.

## How to do it

**Figure 5.**
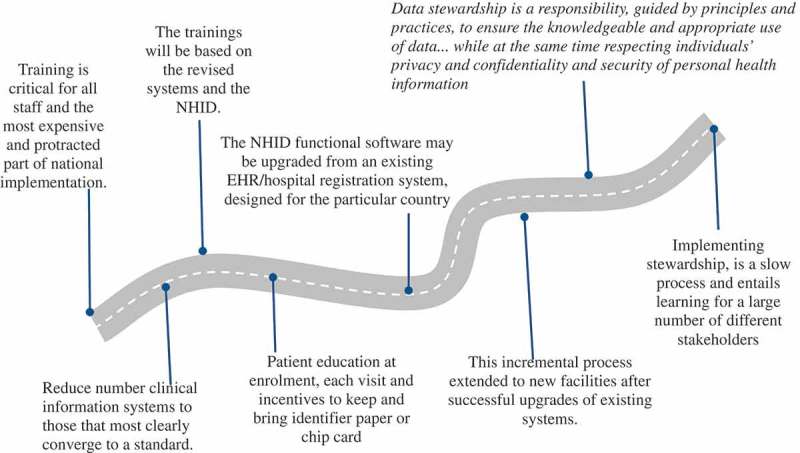
Training requirements, upgrades and data stewardship.

*Training* is critical for all staff, and it may also be the most expensive part of the national implementation (Figure 5). If only some staff are trained, this can potentially lead to difficulties that will affect the success of the implementation process. In a country with many disparate and incompatible clinical information systems, the training process may be very protracted. The introduction of such new technology will require the evaluation and potential redesign of workflow in the site. The training provided needs to be done within the context of the amended workflow. The move to using a NHID is likely to require a paradigm shift in many facilities from recoding services in an aggregated fashion to systematically recording each patient encounter.

The trainings will be based on the revised systems and the NHID. The country will need to decide whether:

- the performance of the clinical system users whose work is what gives the NHID meaning and value, and whose support for the system implementation is essential
- the performance of donors and politicians and champions and visionaries keep all focused on the vision for 5 to 10 years
- implementation will need to include full staff training, retraining and, for some, certification in searching for patients using unique identifiers and quasi-identifiers. The extent of searching prior to issuance of every new unique identifier needs to be logged and used for quality control. A failure to look and find results in the production of a duplicate record, a discontinuity of patient care, double counting of patients served and a reduction of quality of care indices
- apart from training clinic staff, it should also include training of data analysts that can use the information for service improvement.

Patient education needs to be performed at enrolment, reinforcement at each visit, and incentives provided to ensure that they bring and keep the identifier that is used either a barcode or identifier chip card. Although not essential, and more expensive than the barcode, an identifier chip card can also function as storage of limited medical data, an additional benefit (Box 4).

**Box 4.** Potential benefits of identifier chip cards.

Patients carry and control their own basic medical information, and assurance of best practice information through information driven care. People with a chip card register for visit with card swipe, are served first to improve carry rates. The card has a problem list, reasons for all visits, provider information, medical history and other summary medical information for best care wherever client goes. The card contains all prescriptions and can be filled at a pharmacy. The card has all laboratory order requests and results, and vital signs. The card has all diagnoses and treatments, and can prevent harm from conflicting treatments. If a card is lost, place of last care can provide a full copy. It can provide a patient a review of his/her own record.

A few countries have put their full primary care EHR on the chip card in lieu of moving from Tier 2 to Tier 3 facilities. This provides a very robust continuity of care model that works when internet or facility information retrieval fails, since the patient has an copy of his/her EHR.

Disincentives, if client forgets their identifier token or smart card, could include:

- need to queue for process of full re-enrolment prior to clinic visit, if a search fails;
- if a card is lost twice, may need to pay a nominal replacement fee thereafter.

Training in the use of identifier documents at enrolment is a necessity. Photographs or fingerprints can be used and it is necessary to train staff how to take photo and fingerprint biometrics. Fingerprinting may deter drug seeking, and reduce duplicate identifiers’ issuance but taking photographs is easier and less expensive. If neither options are available, the software should allow for robust matching algorithms, or just scan a barcode.

*Deployment*. The NHID functional software deployment may be an upgrade from an existing EHR or hospital registration system, which previously used different identifiers and now uses the NHID designed for the particular country. Software needs to be considered that is purpose-built for, being a population-based EHR with MPI functionality, ensuring that all the speed, interoperability, and patient search and match criteria are met. Deployment should be an incremental process. Only after upgrades of existing systems to use the new NHID have been performed are proceeding well, with the support of available resources, and the upgrade deployment has been successful, would it be a good time to consider moving the NHID and EHR system into new facilities. This process depends on the systems being addressed; in some cases merging records from existing systems could be a very feasible option and will assign new NHID to old records whilst linking the old identifier to the new NHID. This allows old identifiers to be phased out and should also be criteria for the MPI. The process of a new site deployment includes some work that may have been done during assessment, but may need to be revisited:

- Reassess the physical environment – is there a secure room for locking up the system computer(s)? What is the reliability of the power, is there an UPS, generator set, or solar fail-over option for power? Are there any telecommunications?
- Reassess logistics – does the site have transport? What is the distance to nearest site with the system?
- Reassess the adequacy of the staff for the new technology: is the facility understaffed? Do staff rotate through other facilities that are or will be using the system? Have they used a computer before? How long has it been since the staff were trained and do they need to be retrained?

## Follow up – institutionalizing and evolution

Data stewardship is a responsibility that is guided by principles and practices, to ensure the knowledgeable and appropriate use of data. More specifically, stewardship of health data recognizes the benefits to society of using personal health information to improve understanding of health and healthcare, while at the same time respecting individuals’ privacy and confidentiality [29].

Implementing stewardship is a progressive process over time and entails learning for a large number of different stakeholders. It has visible and concrete manifestations, including laws that spell out the protections accorded to personally identifiable information, and the proper uses that can be made of it. It also has invisible and subtle manifestations, such as reducing stigma and discrimination.

A national data repository is a national public good and, if correctly set-up and protected, is a benefit based on the process of aggregating individual health histories. The personal health information that this is based on will need protection through privacy policies that are implemented, and protect the confidentiality and security of that information. Country guidance on protecting personal health information has been developed [9] which countries can adapt, adopt and implement to suit local conditions. Furthermore, an Assessment Tool, Manual and Workbook to assess the existence and implementation of national guidance to protect personal health information have been developed for health facilities, data warehouses and repositories, and national levels [30–32].

Local computers, networks and data repositories have become major targets for political and criminal activities. Recent political activities involved the hacking of the 2016 US and 2017 French elections [33,34] and the 2016 UK Referendum on membership of the European Union [35]. The 2013 hacking of Yahoo [36], the 2016 leaking of information from the Chinese National Center for AIDS/STD Control and Prevention [37] and the mayhem caused by the ransomware cyber-attacks of May and June 2017 [38,39] are just some examples of criminally inspired cyber-attacks. The *Wannacry attack* of May 2017 affected many organizations across the world, including the UK National Health Service [38]. One expert was quoted that ‘*a “massive” increase in spending is needed to prevent another “avoidable” cyber-attack on NHS computer systems’* [40]. While these occurrences should not deter the development and implementation of digital systems, it does reiterate the fact that notions of the confidentiality and security of personal health information need to be taken more seriously than they have been to date in many parts of the world.

The implementation of the stewardship of local and national data repository includes the incremental development and implementation of regulations as the execution of the stewardship proves itself technically and administratively capable and sufficiently autonomous. Relevant laws or regulations need to stipulate the formulation and independence of data repositories with relevant stewardship, and over time it will be apparent if they can protect it from misuse or abuse. Implementing the actual physical stewardship of valuable data is a highly technical endeavour [9], but when properly performed the benefits will outweigh the costs and risks. One needs to evaluate one’s technical capacity to first maintain security, and, second, to determine and maintain appropriate access.

Start slowly but initially focus on security. Once achieved, one can then make transparent accommodations of requests for information with ethics boards approval and consented studies. Via an open process, the ‘public use data’ can be defined to get the information used for the common good. Over time the number of users and the types or sensitivity of data released may increase, in a transparent manner, while monitoring outcomes and being sensitive to the level of public concern. Denmark is an example of a country that that has made a lot of progress in this area [41].

However, implementation and ensuring the confidentiality and security of personal information remains an ongoing issue, as was recently demonstrated in the problems faced by Sweden. Sweden experienced an ‘extremely serious’ security breach, ‘which followed a 2015 data outsourcing contract between the national transport agency and IBM Sweden’ in which security clearance requirements for foreign information technology workers were waived when signing the agreement, in breach of privacy and data protection laws” [42]. Swedish media reported that besides the ‘entire national driver’s license database, the records potentially included information on intelligence agents, military and police transport and personnel, people with criminal records and those in witness protection programs’ [42]. This event highlights the even greater need for protecting personal information, if personal health information can be tracked across different social sectors.

*Institutionalization*: institutionalizing these processes should begin at the earliest stages of the assessment process. Some reflect the understanding of national values as developed during the consensus meetings and reassessments. Others reflect a maturing of society’s understanding of shared benefits and associated risks and the need for tolerating differences to achieve the largest population benefits. The points described in Box 5 provide indications of public and political will, and manifestations at systems’ level.

**Box 5.** Understanding of shared benefits, risks and actions.

***Reflections of public and political will*** a broad and correct understanding of the system, its purpose, benefits and risks; broad popular support and trust of the system among civil society and all stakeholders. the existence of laws that support the system’s purpose and necessary procedures. laws, regulations and procedures that assure the safety of the system, that it protects the confidentiality and security of personal health information, that it is trusted and practical, and undergoes regular appraisals of its security. that there is a mechanism that assures the system will remain well-resourced long term. that a plan exists for decommissioning confidential information and tools, if the need exists.
***Manifestations at the level of the system*** the existence of feedback control processes for all critical indicators of system operation, which provide information about the integrity of each of the elements of the system components and processes. providing this information in timely and actionable form to the persons responsible for any necessary interventions, and keeping alternative responders and supervisor abreast of developments. feedback control processes that are designed to maintain the homeostasis of certain operations at the correct balance, and in other cases are designed to optimize performance over time through ever refined system processes such as match algorithms. learning on the part of all persons that are part of the system, including not forgetting identity cards, so that the dependence on the matching algorithm is continually reduced. growing numbers of vital private and public health functions that are served by the system, in accord with wise and fair data use policies that both serve the public interest and continue to assure the security of the individual interest. For instance, the necessity for highly personalized cancer care or early interventions in disease with genetic markers may become standards of care, and the life-long records prove life-lengthening. that everyone carries their identity card, accepts a couple of biometrics, and appropriately trusts the data stewards with sensitive information where public interest has exercised exception to individual rights. Given the protective measures in place – including safety, justice, confidentiality and security of the system – and that these have met with public approval. that in the public realm the automated summaries of individual records to produce a national aggregated resource of information are demonstrated to be practical with reasonable precautions and controls. The pooled information can be used for training, education and research to develop public policies, with an ever growing use of such a valuable national information asset. the existence of interoperability at all levels of the system, between the potentially disparate clinical information systems which have evolved into standardized systems to provide continuity of information to the users of the service. that patient care can rely on the system and benefits from it, as do government and academic institutions and that business may profit by it, provided this is consented, the personal health information is protected and the work serves the wider public good.

For this process to be successful, it needs to have a long-term perspective, as long as personal health information needs to be in the public domain in order to improve public health, by harnessing the synergies of the different information streams based on the life-long health experiences of each person in the country.

Implementation is more of a political process than an engineering one and as such this process is never completed as technologies and political ideologies may change. Much of its success hinges on human will and performance:

- the performance that the patient carries his/her card and trusts the system
- the performance of the system registration staff who educate and reinforce the client
- the potentially life-saving tasks of preventing duplicate and overlaid health records, preventing misidentification that remains a leading cause of death [43]
- the performance of the many types of ICT and informatics staff who provide ongoing back-up maintenance and continuously upgrade the system security, retune the MPI algorithms as the database grows and changes, and continually assess risks
- the maintenance of all the elements by the staff of a secure national system, serving millions of users in a manner that maintains their trust
- that staff develop and maintain expertise in many types of hardware and equipment, devise and implement automated feedback control systems for every vital element of the national systems operation.

An example of how a national health system has become institutionalized can be seen in the UK. The UK NHS was established in 1948 and was based on a pre- and post-World War II political consensus that every UK citizen should have access to healthcare irrespective of who they are and where they are, and that was free at point of service provision [44]; public discussion about the need to develop an NHS goes back to the early 1900s. This taxation-based system has proved to be very popular and, despite many technological changes, changes in expectations of users of services and political ideologies since its foundation, every government has declared its intention to maintain and improve the NHS. Recent polls among UK citizens indicate that *‘There is strong support for the principles of the NHS across all sections of British society. Of those surveyed, 89% agree that the government should support a national health system that is tax funded, free at the point of use and provides comprehensive care for all citizens’* [45]. Nevertheless, the NHS has faced recurrent problems, there have been endless reorganizations, paying tribute to the notion that systems like the NHS will continue to evolve, as technologies and expectations by users and providers of services evolve and result in changing societal attitudes.

## Conclusion

The development of individual longitudinal linked medical records across health services and the use of de-identified personal health information to monitor, evaluate and improve services on a continuous basis are going to be greatly aided by the development of NHIDs. For this process to be optimally successful it needs to be a progressive multi-stakeholder process that involves identifying champions to ‘carry the torch’ and highlight evidence of improvement during this medium- to long-term process. These ‘champions’ need to be representatives from different communities that have a stake in ensuring this process is not only technically successful but that it conforms to and is in line with accepted human right principles [46]. Champions should include members of the technical community, healthcare professionals, representatives from patient organizations and other relevant civil society organizations, academics, private investors and politicians from across the political spectrum. The focus should not only be on technical issues but provide ongoing evidence of improving service delivery through the use of this information. The technical aspects for the development and implementation of a NHID system are less of an issue compared with the social, economic and cultural aspects of these processes. It is these factors that will ultimately determine whether the process is successful or not.

## Paper context

Linking data from different sources enables longitudinal individual health records to be constructed and de-identified personal health information (PHI) can be used for monitoring and evaluating health services. National health identifiers (NHIDs) enable such linkages and this paper provides practical guidance for developing and implementing NHIDs. Success of this process is ultimately dependent on social, political and economic factor rather than technical ones, and protecting the confidentiality and security of PHI.
